# Annotations

**Published:** 1897-05-22

**Authors:** 


### Annotations.

### Sanitation and Morality.

The subject of "The Health of the Army in India" has again been brought into prominence by the striking speech of Lord Roberts in the House of Lords last Friday, supported by that delievered by Lord Lister on Monday. We feel strongly that this is a matter in regard to which every medical man should definitely make up his mind, and that in doing so he should have regard to medical rather than moral considerations. A strong attempt has been made to discuss the question as if it were merely one of morality, and to tar with the brush of moral turpitude all who venture to hint that we should take some care to protect the health of the young soldiers whom we send out to defend our Eastern possessions, and whom for that purpose we expose to no ordinary temptations. It cannot, then, be too strongly insisted on that, primarily and in essence, this is a question of medicine and sanitation, and that medical and sanitary authorities should have the first say upon it. We do not pretend that points of morality are not involved, but that is another affair. The current morality of every country should find expression in its laws, and although we would be prepared to go a long way with those who would bring into our laws some modification of the sanction which they at present give to free and unrestricted vice, we cannot but feel that it is a scandal that sanitary science should be held back from interference with disastrous diseases which spring from practices which, however immoral, the law allows. Circumstances are constantly arising in daily life in which it is impossible to avoid encouraging in an indirect manner practices which one regards with the gravest possible dislike, and this is one of them. When we buy cheap shirts we encourage "sweating," and when we buy wax vestas we encourage phosphorus poisoning. We none the less, however, buy both vestas and cheap shirts; but at the same time we pass laws and send inspectors to prevent the grinding of the poor and the poisoning of the match-makers. And so in regard to these diseases as encouragers of vice. If it is true that the moral sense of the community is outraged by the vice which the prevalence of these diseases shows to be so common, let our legislators intervene to stop it. But that is quite another story, as our legislators would soon find out. At present what we have to do is to stop the spread of the disease, which ought not to be beyond the powers of sanitary science aided by a reasonably efficient police.

### The History of a Bruise.

Everyone who knows anything about lunacy knows how liable lunatics are to show upon their persons signs of bruising. Hence, it becomes of extreme importance, in the investigation of any case in which suspicions arise of violence having been used, to be able to ascertain whether the physical signs of bruising which are found fit in with the tale told of the mode of its occurrence. Dr. T. Claye Shaw has recently investigated the history of 111 bruises, making inquiries as to the time at which the discolouration appeared, and observing the colour which it presented, and the time and mode of its disappearance. In a few cases, perhaps 5 per cent., the bruising showed itself within six hours of the injury, in some within an hour; but, taking them by days, it came out that 25 per cent. appeared the same day, 64 per cent. the next day, 13 per cent. 2 days later, and 2 per cent. 3 days later. As regards the time of disappearance of the mark there were very great differences. One very slight mark disappeared in 1 day; 14, in 4 days; 13, in 6 days; 13, in 8 days; 4 lasted 11 days; 1, 18 days; 3, 22 days; 1, 34 days; and 1 lasted 39 days. It thus appears that 50 per cent. of the bruises disappeared in from six to nine days. In regard to colour, many very interesting details are given, which may be summed up thus: Mixtures of red and violet are rarely seen pure after the third day; green comes mixed with the above about the second or third day; the mixed green and yellow begins about the third day and lasts to the sixth or seventh; and the pure yellow is always the last. If a person shows much purple or red in the bruising we may fairly say that the injury was done within the last four days; if it is yellow-red or greenish yellow, then it has been done about four or five days; and if yellow, it certainly has not been done within four days.

### American Action in Regard to Dispensary Abuse.

The problems of hospital abuse and hospital reform have gone through many and various phases, but nowhere, so far, has the nettle been grasped with such firmness as the Legislature of New York State proposes to do in a Bill which has recently passed both Houses, and now only awaits the signature of the Governor to become law. There is in New York a State Board of Charities, but the commissioners do not yet possess power to close any dispensary which in their opinion may not be fulfilling the object for which it was founded. By the new Bill, however, all dispensaries, whether of a municipal or private character, will be subject to the supervision and control of the State Board of Charities, which body is empowered to "annul the corporation, or suspend the operations, or revoke the licence of any dispensary for wilful neglect or failure on the part of its managers, officers, or employés to comply with the rules and regulations" established by it. Now it is stated in the last report of this board that dispensaries have rapidly increased, that they institute no proper inquiry as to the circumstances of the patient, and that "it is very evident that when any so-called charities have so far departed from their original objects and purposes as to invite or even allow persons of wealth or moderate competence to enjoy their benefits, they have ceased to fulfil their proper functions and should be suppressed." This is going to the root of the matter. But we ask ourselves whether this is not rather like burning the house down to roast the pig.. Surely patience and common sense should be able to reform abuses without suppressing charities. The evil of such drastic laws as the one here spoken of lies in the fact that, although it is quite out of the question to believe that it will ever be generally put in force, it places enormous power in the hands of the State Board, power which may be brought into play for purposes not at all intended by the framers of the law; for what charity is there that does not sin sometimes, and that might not be threatened with closure at any moment?

